# Micronutrient Deficiencies in Nepalese Adolescents: National Prevalence and Associated Factors

**DOI:** 10.1002/fsn3.71629

**Published:** 2026-03-11

**Authors:** Kingsley Emwinyore Agho, Stanley Chitekwe, Lucy Ngaihbanglovi Pachuau, Biniyam Sahiledengle, Sanjay Rijal, Naveen Paudyal, Sanjeev Kumar Sahani, Andre Renzaho

**Affiliations:** ^1^ School of Medicine Western Sydney University Campbelltown New South Wales Australia; ^2^ Faculty of Health Sciences University of Johannesburg Johannesburg South Africa; ^3^ Translational Health Research Institute (THRI) Western Sydney University Penrith New South Wales Australia; ^4^ Nutrition Section United Nations Children's Fund (UNICEF) Ethiopia Addis Ababa Ethiopia; ^5^ School of Health Sciences Western Sydney University Campbelltown New South Wales Australia; ^6^ Department of Public Health Madda Walabu University Goba Referral Hospital Bale‐Goba Ethiopia; ^7^ United Nations Children's Fund (UNICEF), United Nations (UN) House, Pulchowk Kathmandu Nepal

**Keywords:** adolescent, boys, folate, girls, micronutrients, Nepal

## Abstract

Micronutrient deficiencies, including iron, vitamin A, and folate, remain a major public health among adolescents in low‐ and middle‐income countries, including Nepal. Understanding the prevalence and factors associated with deficiencies is essential for designing targeted nutrition interventions. Data from 1025 boys and 1852 girls ages 10–19 years were extracted from the 2016 Nepal National Micronutrient Status Survey (NNMSS) to examine the prevalence and factors associated with ferritin, soluble transferrin receptor (sTfR), vitamin A, and red blood cell (RBC) folate status among adolescent boys and girls. All micronutrient outcomes were categorized as binary (1 = if deficiency and 0 otherwise). The prevalence of RBC folate deficiency among adolescent girls was 16.2% and other micronutrient deficiencies were higher in girls compared with boys, ferritin (18.0% vs. 4.8%), sTfR (13.8% vs. 12.2%), and vitamin A (4.4% vs. 2.8%). Multivariable analysis indicated that dietary factors, region, and low and normal Body Mass Index (BMI) were significantly associated with micronutrient deficiencies. Adolescents who did not consume eggs had higher odds of iron deficiency (boys: AOR: 4.35, 95% CI: 1.24–15.24; girls: AOR: 3.15, 95% CI: 1.48–6.69). Girls from the far‐western region or with low and normal BMI were more likely to be iron‐deficient. Eastern‐region girls had higher odds of folate deficiency, and those not consuming nuts or seeds were more prone to vitamin A deficiency. This study found that adolescent boys and girls in Nepal experience a substantial burden of micronutrient deficiencies, with girls disproportionately affected. These findings highlight the need for sustainable and equitable adolescent nutrition programs in Nepal.

## Graphical abstract

This study found that consumption of eggs is associated with iron deficiency among adolescent boys and girls in Nepal. Adolescent girls from the eastern region are more likely to have a low ferritin level. Being iron deficient (sTfR levels) was significantly higher in adolescent girls from the Janajati caste group.

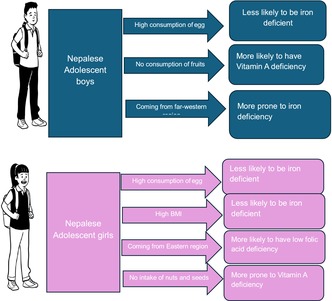

## 1 Introduction

Globally, an estimated 2 billion people are affected by essential vitamins and micronutrients, with disproportionate effects on vulnerable populations, including adolescents (Bailey et al. 2015). Adolescents are often considered less nutritionally vulnerable than pregnant and lactating women as well as young children and are therefore frequently excluded from nationally representative nutritional assessments (Woodruff et al. 2006). However, evidence suggests that adolescents are at significant risk of deficiencies in key micronutrients such as iron, vitamin A, and folate.

Vitamin A deficiency, the most common micronutrient deficiency worldwide, is prevalent among adolescent girls and boys (Horwitz et al. 1998). Vitamin A plays a critical role in vision, immune function, and overall health, and its deficiency is associated with increased morbidity and mortality from respiratory and gastrointestinal infections (Gorstein et al. 2003). Iron deficiency among adolescents has been shown to negatively affect learning, memory, attention, academic achievement, and physical performance in both sexes (Beard and Connor 2003; Beard 2001; Grantham‐McGregor and Ani 2001). Inadequate folate intake can rapidly lead to deficiency, which may result in anemia and, during pregnancy, neural tube defects (Healthline 2016). Although folate is available from natural food sources and fortified foods, synthetic folic acid supplementation is commonly recommended for women of reproductive age including adolescent mothers to prevent deficiency (Healthline 2016; Iyer and Tomar 2009). Low folate levels have also been associated with depression later in adulthood, highlighting its broader relevance to mental health (Coppen and Bolander‐Gouaille 2005).

Previous studies have identified inadequate dietary diversity as a major contributor to micronutrient deficiencies, including folate, vitamin A, and iron, among women in Nepal (Henjum et al. 2015). and these deficiencies may have originated during adolescence. Given the importance of adequate iron stores for adolescent girls' future reproductive health and the impact of iron and folate deficiencies on physical, cognitive, and mental health outcomes (Beard 2001; Grantham‐McGregor and Ani 2001; Coppen and Bolander‐Gouaille 2005) Hence, it is critical to examine the prevalence and determinants of these deficiencies during adolescence.

There have been several studies that examined iron deficiency anemia among adolescent girls and boys in Nepal (Ford et al. 2022; Baral and Onta 2009; Baral 2003; Rikimaru et al. 2003; Tiwari 2000; Regmi and Adhikari 1994; Kanodia et al. 2016; Sinha et al. 2012), evidence on deficiencies of other micronutrients and biomarkers of anemia such as ferritin, soluble transferrin receptor (sTfR), and red blood cell (RBC) folate remains limited. One study assessed iron and vitamin A status among Nepalese adolescents but was restricted to refugee populations and did not examine associated factors (Woodruff et al. 2006). Consequently, there is a lack of recent, population‐based nationwide representative evidence on factors associated with multiple micronutrient deficiencies among adolescent boys and girls in Nepal. Findings from this study will help inform nutrition policies and programs aimed at identifying and addressing micronutrient deficiencies among high‐risk adolescent subpopulations.

## 2 Methods

### 2.1 Data Source

The data source for this study was the 2016 Nepal National Micronutrient Status Survey (NNMSS).

### 2.2 Survey Design and Participants

The NNMSS is cross‐sectional, population‐based, and nationally representative in design. The survey was a stratified multistage cluster sampling to provide representative estimates for the five development regions in the country, namely, Eastern, Central, West, Midwest and Far West; and of the three ecological zones of the entire country, namely, Terai (plains), Hills, and Mountains. In the first stage, 180 clusters were selected (75 each in Hill and Terai and 30 in Mountain). After the clusters were selected, clusters with more than 300 households were considered large clusters and were further divided into approximately 100 households each in the next stage. Within each selected cluster, 24 households were selected systematically, resulting in a total of 4320 households (180 clusters × 24 households). Eligible individuals from these 24 households in each cluster who belong to the survey population groups were selected. For adolescent boys and girls 10–19 years, a total of 1045 adolescent boys 10–19 years were interviewed, and 1025 responded, yielding a response rate of 98.1%, while a total of 1898 adolescent girls 10–19 years were interviewed, and 1865 responded, yielding a response rate of 98.3%. Details of the NNMSS can be obtained elsewhere (Ministry of Health Nepal, New ERA, UNICEF, EU, USAID, CDC 2017). In the analysis, 13 pregnant adolescent girls and three adolescents with uncertain pregnancy status were excluded, resulting in a final analytical sample of 1850 adolescent girls (World Health Organization 2011a, 2011b, 2015).

### 2.3 Data Collection

Administered questionnaires were used to collect information on each household and for adolescent boys and girls aged 10–19 years. Venous blood samples were collected from all eligible participants, as well as urine and stool samples. Household questionnaire was used to collect information from households and all study participants. The household questionnaire was used to identify all members of the household who were eligible to participate in the survey (i.e., adolescent boys and girls aged 10–19 years). The adolescent boys' questionnaire was used to collect information on sociodemographic characteristics; participation in key national nutrition and other interventions; recent micronutrient supplementation intake (zinc, iron, folic acid, vitamin A, multiple micronutrients); dietary diversity scale; smoking habits; 2‐week recall of fever, cough, and diarrhea; and anthropometric measures. The adolescent girls' questionnaire was used for a similar purpose. Details of data collection has been reported elsewhere (Ministry of Health Nepal, New ERA, UNICEF, EU, USAID, CDC 2017).

### 2.4 Outcome Indicators

The outcome variables examined in this study were ferritin, sTfR, vitamin A, and RBC folate which were informed by data from the 2016 NNMSS and Zinc and B12 was not measured for adolescents due to public health relevance, feasibility, cost, and available laboratory capacity (Government of Nepal Ministry of Health and Population U, USAID, European Union, CDC, New Era 2018). NNMSS was the first nationally representative survey to comprehensively assess micronutrient status among adolescents in Nepal (Government of Nepal Ministry of Health and Population U, USAID, European Union, CDC, New Era 2018). Iron status was assessed using inflammation‐adjusted serum ferritin and sTfR concentrations, with iron deficiency defined as ferritin < 15 μg/L and tissue iron deficiency defined as sTfR > 8.3 mg/L (World Health Organization 2011a; Government of Nepal Ministry of Health and Population U, USAID, European Union, CDC, New Era 2018) Folate status was assessed using RBC folate concentrations, with deficiency and insufficiency classified using Institute of Medicine and WHO thresholds, including cutoffs indicating folate‐deficient anemia and levels insufficient for neural tube defect prevention (World Health Organization 2015; Government of Nepal Ministry of Health and Population U, USAID, European Union, CDC, New Era 2018) and RBC folate deficiency was classified as RBC folate < 305 expressed in nanomoles per liter (nmol/L). Vitamin A status was evaluated using retinol‐binding protein (RBP), calibrated to serum retinol, with deficiency defined as RBP < 0.64 μmol/L, equivalent to serum retinol < 0.70 μmol/L (Government of Nepal Ministry of Health and Population U, USAID, European Union, CDC, New Era 2018). For the purposes of statistical analyses, each micronutrient outcome was categorized as a binary variable, coded as “Yes” for deficiency and “No” otherwise.

In the 2016 NNMSS data, inflammation was assessed using serum C‐reactive protein (CRP) and α‐1‐acid glycoprotein (AGP). To account for the influence of inflammation on micronutrient biomarkers, the BRINDA regression adjustment approach was applied. Natural log–transformed biomarker concentrations were regressed on log‐transformed CRP and AGP values. Adjusted biomarker concentrations were then estimated by subtracting the predicted inflammatory effects and standardizing values to reference concentrations representing minimal inflammation (CRP and AGP at their respective lowest deciles). Individuals with missing CRP or AGP measurements were excluded from the adjusted analyses. This approach follows established BRINDA guidelines and methods was reported in detail in the 2016 NNMSS (Government of Nepal Ministry of Health and Population U, USAID, European Union, CDC, New Era 2018).

### 2.5 Potential Confounding Variables

Previous literature on factors associated with micronutrient deficiency informed the choice of possible confounding variables (Balcı et al. 2012; Alzaheb and Al‐Amer 2017; Alaofè et al. 2017; Harding et al. 2018; Agho, Chitekwe, Sahiledengle, et al. 2024; Agho, Chitekwe, Rijal, et al. 2024; Chitekwe et al. 2022) and their availability in the Nepal National Micronutrient Survey dataset. We categorized these variables into individual‐level factors, household‐level factors, communitylevel factors, anthropometric factors, health status factors, and diet habits (a day before the survey).

The individual‐level factors included the participant's education level and marital status; household‐level factors included the household wealth index and ethnicity. The household wealth index was represented as a score of household assets through the principal components analysis method (PCA) (Filmer and Pritchett 2001). Scores were assigned to each de jure household member after the index was computed, ranking each sample member by their score. In this study, the wealth index was categorized into five quintiles: poorest, poorer, middle, rich, and richest. Community‐level factors included geographical region and ecological zone. Anthropometric factors included the participants' mean weight, mean height, mean body iron stored (BIS), and body mass index (BMI); health status factors included fever, cough, and diarrhea in the last 2 weeks; and diet habit factors included the participants' consumption of the different food groups. The potential confounding variables used in this study have been described elsewhere in different populations (Agho, Chitekwe, Sahiledengle, et al. 2024; Chitekwe et al. 2022).

### 2.6 Statistical Analysis

The statistical analysis approach for this study was guided by the binary nature of the outcome variables, which indicated ‘1’ for “Yes” for deficiency and ‘0’ for “No” otherwise and by the complex survey design of the 2016 NNMSS. All analyses accounted for the multistage sampling design of the NNMSS, including clustering, stratification, and sampling weights, to ensure nationally representative estimates (Government of Nepal Ministry of Health and Population U, USAID, European Union, CDC, New Era 2018).

Descriptive analyses were conducted to characterize the study population and to estimate the prevalence of micronutrient deficiencies among adolescent boys and girls. These analyses were performed using the survey (svy) commands in Stata version 17 (StataCorp, College Station, TX, USA) to appropriately adjust for clustering and stratification when estimating proportions and corresponding 95% confidence intervals. The epctile command was used to compute interquartile ranges for continuous variables.

Bivariable logistic regression analyses, adjusted for survey design and sampling weights, were conducted to estimate unadjusted odds ratios (ORs) for associations between selected covariates and each micronutrient deficiency outcome, including ferritin, sTfR, vitamin A deficiency, and RBC folate.

To reduce statistical bias and improve model stability, only micronutrient deficiency outcomes with a prevalence greater than 4% were included in the multivariable logistic regression analyses because logistic regression models perform poorly due to small sample size (Vittinghoff and McCulloch 2007). In the analysis, confounding variables with a *p*‐value < 0.20 in the bivariable analyses were retained and used in the multivariable regression analyses and these confounding variables were residence, state, age, level of education completed, ecological zone, height, weight, BMI, BIS, consumption of vitamin A–rich fruits, other vegetables, meats, eggs, legumes, and nuts. Using *p*‐value < 0.20 to select confounding variable to be included in the multivariable logistic regression analyses aligns with standard epidemiological modeling practices and helps ensure that potentially important confounders are retained in the multivariable models (Heinze and Dunkler 2017; Hosmer et al. 2013). After retaining the potential confounding factors, a manual backward elimination procedure was applied for multivariable logistic regression analysis to remove nonsignificant variables (*p* > 0.05) because of weighted data analysis and to determine the adjusted odds ratios of micronutrient deficiencies including ferritin levels, sTfR, RBC folate and vitamin A. We tested for any colinearity in the final model and reported the results. The associated factors were presented as the adjusted odds ratio (95% CI) for the variables retained in the final step.

### 2.7 Ethical Consideration

Written informed consent was obtained from respondents who could read, while oral informed consent, coupled with the signatures of witnesses, was obtained from those who could not read. Regarding those who could not read, the interviewer read out the informed consent to each of them, who then proceeded to mark the consent form before commencing the survey if the consent was given. The respondents were assured of maintaining the privacy of their personal information, which was to be used only for the study. Signed consents were also obtained from participants aged 18 years and older for an interview and biological specimen collection. Respondents were offered gifts that included a towel, toothpaste, toothbrush, soap, nail cutter, and 1 kg of iodised salt as an incentive for participation. Ethical clearance for NNMSS was given by Nepal Health Research Council (NHRC) on 18th February 2016.

## 3 Results

### 3.1 Characteristics of the Study Participants

Table 1 summarizes the demographic and socioeconomic characteristics of the adolescent boys and girls included in the study. The table showed that the percentage and count of the weighted and unweighted data were similar (see Table 1 for details), indicating that adjustment for clustering, stratification, and sampling weights to obtain nationally representative estimates would not introduce statistical bias.

**TABLE 1:** Distribution of characteristics of Nepalese adolescent boys and girls aged 10–19 years.

Characteristics	*N*	N* (%*)	*N*	N* (%*)	Characteristics	*N*	N* (%*)	*N*	N* (%*)
	Boys (N* = 1025)		Girls (N* = 1852)			Boys (N* = 1025)		Girls (N* = 1852)	
**Community level factors**					**Anthropometry#**				
*Residence*					Mean weight (sd)	34.2 (19.0)	32.8 (19.2)	39.7 (10.1)	39.5 (9.9)
Urban	143 (13.9)	142 (13.6)	215 (11.6)	188 (10.1)	Mean height (sd)	150.3 (15.2)	150.7 (15.3)	146.5 (10.0)	146.6 (9.8)
Rural	882 (86.1)	883 (86.4)	1635 (88.4)	1664 (89.9)	*Body iron stored (BIS), (mg/kg)*				
*State*					Mean BIS (sd)	5.9 (2.6)	5.8 (2.7)	4.3 (3.4)	4.2 (3.4)
Province 1	179 (17.3)	168 (16.4)	301 (16.3)	325 (17.1)	*BMI (kg/m* ^*2*^)				
Province 2	106 (10.3)	233 (22.7)	201 (10.9)	395 (20.8)	≤ 18.5	633 (60.1)	634 (61.9)	933 (50.4)	962 (52.1)
Province 3	132 (12.8)	174 (17.0)	206 (11.1)	319 (16.8)	19–25	376 (36.0)	367 (35.8)	871 (47.1)	843 (45.6)
Province 4	102 (9.9)	99 (9.5)	187 (10.1)	193 (10.2)	25+	16 (1.9)	24 (2.3)	43 (2.3)	44 (2.4)
Province 5	205 (20.4)	192 (19.1)	378 (20.4)	354 (18.7)	**Health status** ^#^				
Province 6	87 (8.6)	44 (4.4)	164 (8.9)	99 (5.2)	*Had fever*				
Province 7	214 (20.7)	114 (11.0)	413 (22.3)	212 (11.2)	Yes	130 (12.4)	111 (10.8)	279 (15.1)	277 (15.0)
*Geographical region*					*Had cough*				
Eastern	208 (20.2)	232 (22.7)	354 (19.1)	417 (22.4)	Yes	143 (13.7)	121 (11.8)	352 (19.0)	339 (18.3)
Central	209 (20.3)	342 (33.4)	354 (19.1)	596 (32.2)	*Had diarrhea*				
Western	195 (18.9)	204 (19.9)	350 (18.9)	374 (20.2)	Yes	71 (6.7)	71 (7.0)	155 (8.3)	158 (8.6)
Mid‐western	199 (19.9)	133 (12.9)	379 (20.4)	255 (13.8)	**Diet habit (a day before the survey)**				
Far‐western	214 (20.7)	114 (11.1)	413 (22.3)	210 (11.4)	*Consumed roots or tubers/starchy foods*				
*Ecological zone*					Yes	827 (79.1)	839 (81.9)	1491 (80.6)	1504 (81.2)
Mountain	157 (15.6)	70 (7.0)	289 (15.6)	137 (7.4)	*Consumed legumes and nuts*				
Hill	435 (42.0)	429 (41.4)	778 (42.1)	811 (43.8)	Yes	726 (69.5)	731 (71.3)	1305 (70.5)	1287 (69.5)
Terai	433 (42.4)	526 (51.6)	783 (42.3)	904 (48.8)	*Consumed nuts and seeds or any food from nuts or seeds*				
**Household level factors**					Yes	65 (6.2)	76 (7.4)	120 (6.5)	129 (7.0)
*Household Wealth Index*					*Consumed dairy products*				
Poorest	252 (24.1)	191 (18.6)	491 (26.5)	420 (22.7)	Yes	444 (42.5)	456 (44.5)	715 (38.6)	725 (39.1)
Poorer	211 (20.2)	208 (20.3)	426 (20.0)	409 (22.1)	*Consumed eggs*				
Middle	209 (20.0)	227 (22.1)	338 (18.3)	372 (20.1)	Yes	125 (12.0)	138 (13.4)	189 (10.2)	185 (10.0)
Richer	165 (15.8)	179 (17.5)	322 (17.4)	350 (18.9)	*Consumed liver, kidney, heart or other organ meat or blood*				
Richest	188 (18.0)	221 (21.5)	273 (14.8)	301 (16.2)	Yes	80 (7.7)	72 (7.0)	107 (5.8)	103 (5.5)
*Ethnicity (Caste)*					*Consumed other meats (chicken, goat, buffalo, pigs, ducks)*				
Brahmin/Chettri	436 (41.7)	366 (35.7)	705 (38.1)	612 (33.0)	Yes	274 (26.2)	265 (25.9)	441 (23.8)	436 (23.5)
Dalit	159 (15.2)	147 (14.4)	323 (17.5)	303 (16.4)	*Consumed fish (fresh or dried fish, shellfish, prawn, crab etc.)*				
Janajati	301 (28.8)	303 (29.5)	602 (32.5)	611 (33.0)	Yes	58 (5.5)	57 (5.6)	96 (5.2)	111 (6.0)
Others^$^	129 (12.3)	209 (20.4)	220 (11.9)	327 (17.6)	*Consumed other vitamin A‐rich fruits*				
**Individual level factors**					Yes	99 (9.5)	110 (10.7)	839 (45.4)	852 (46.0)
Mean age (sd)	14.0 (2.7)	14.1 (2.7)	14.3 (2.8)	14.3 (2.8)	*Consumed vitamin A‐rich fruits*				
*Level of education completed*					Yes	137 (13.1)	150 (14.6)	143 (7.7)	152 (8.2)
No education	348 (33.3)	363 (34.6)	625 (33.2)	629 (3.0)	*Consumed other fruits*				
Completed primary	553 (52.9)	528 (50.2)	995 (52.8)	948 (51.3)	Yes	417 (39.9)	401 (39.1)	1408 (76.1)	1391 (75.1)
Secondary or higher	144 (13.8)	160 (15.2)	262 (13.9)	272 (14.7)	*Consumed dark green leafy vegetables*				
*Currently married or living together* ^*#*^					Yes	446 (42.7)	430 (42.0)	223 (12.0)	235 (12.7)
Yes	29 (2.8)	29 (2.8)	187 (9.9)	196 (10.6)	*Consumed other vegetables*				
No	996 (95.3)	996 (97.2)	1663 (88.3)	1656 (89.4)	Yes	776 (74.3)	776 (75.6)	771 (41.7)	734 (39.6)
					*Mean food groups (sd)*	4.3 (1.8)	4.3 (1.8)	4.2 (1.7)	4.2 (1.7)

*Note:* $ = Other Terai Caste, Newar, and Muslim; # = 20 observations missing boys and 2–4 observations missing in girls. *N* = unweighted sample; N* = weighted sample, % = unweighted percentage and %* = weighted percentage.

Abbreviations: BMI, body mass index; BSI, body iron stored.

#### 3.1.1 Boys

A weighted sample of 1025 adolescent boys who participated in the study (see Table 1). The mean age of the participants was approximately 14 years. Approximately half of the boys had primary education, with 15% attaining secondary education or higher. The majority of the boys did not contract fever, cough or diarrhea (89.2%, 88.2% and 93.0% respectively). More than three‐quarters of the participants came from rural areas, more than half of them came from the Terai ecological zone, and the majority were from Province 2 (22.7%). While most of the participants consumed starchy foods as well as legumes and nuts (81.9% and 71.3%, respectively), most of them did not consume dairy products, eggs, flesh foods, or fruits and vegetables.

#### 3.1.2 Girls

The total weighted sample size was 1852 adolescent girls aged 10–19 years, and the mean age was 14.3 ± 2.8 years (Table 1). Slightly more than half of the participants (51%) had primary education only, and just 3% had no schooling. A large majority (89%) of them were single. Most of the participants did not have a fever, cough, or diarrhea prior to the survey (85%, 91%, and 82%, respectively). The mean weight and height of the participants were 39.5 ± 9.9 kg and 146.6 ± 9.8 cm, respectively. Additionally, the mean body iron stored (BIS) body weight was 4.2 ± 3.4 mg/kg, and more than half of the participants (52%) had a BMI of 18.5 kg/m^2^ or lower. Most of the adolescent girls (23%) were from the poorest households, while the minority (16%) were from the richest households. Approximately nine in every 10 were from rural areas, and the majority (21%) came from province 2. The majority of the participants consumed starchy foods made from roots or tubers, legumes and nuts, offal, and other fruits (81%, 70%, 95%, and 75%, respectively). Furthermore, most of the participants did not consume dairy products, eggs, other meats, fish, vitamin A‐rich fruits, dark green leafy vegetables, and other vegetables (61%, 90%, 77%, 94%, 54%, 92%, 87%, and 60% respectively). The mean number of food groups consumed was 4.2% ± 1.7%.

### 3.2 Prevalence and Mean Concentrations of Ferritin, sTfR, Vitamin A Deficiency, and RBC Folate Deficiencies

#### 3.2.1 Boys

Table 2 presents the prevalence and mean serum concentrations of selected micronutrients among adolescent girls and boys aged 10–19 years. Among adolescent boys, the mean ferritin concentration was 52.4 ± 29.9 μg/L, with a prevalence of 4.8%. The mean sTfR concentration was 6.6 ± 2.8 mg/L, and 12.2% of boys were classified as having sTfR deficiency. The mean vitamin A concentration was 1.2 ± 0.4 μmol/L, with a deficiency prevalence of 2.8% (Table 3).

**TABLE 2:** Prevalence and mean concentration and distribution of ferritin, sTfR, folic acid/RBC folate and vitamin A status among adolescent girls and boys aged 10–19 years in Nepal.

Micronutrient	Cut‐off values	Mean (sd)	Quartiles			Pr (%)
			Q25	Q50	Q75	
Adolescent girls						
Ferritin (*n* = 1841)	< 15 μg/L	35.2 (22.3)	18.39	30.92	46	18.0
sTfR (*n* = 1841)	> 8.3 mg/L	6.8 (4.5)	4.8	5.7	6.8	13.8
Vitamin A (RBP, *n* = 1841)	< 0.70 μmol/L	1.2 (0.3)	0.9	1.1	1.3	4.4
Folic acid/RBC folate (*n* = 1841)	< 305 nmol/L	499.8 (241.8)	353.7	460.6	593.6	16.2
Adolescent boys						
Ferritin (*n* = 1014)	< 15 μg/L	52.4 (29.9)	31.6	46.6	69.2	4.8
sTfR (*n* = 1014)	> 8.3 mg/L	6.6 (2.8)	5.2	6.0	7.1	12.2
Vitamin A (RBP, *n* = 1014)	< 0.70 μmol/L	1.2 (0.4)	1.0	1.2	1.5	2.8

Abbreviations: Pr, prevalence; RBC, red blood cell; RBP, retinol binding protein; sTfR, soluble transerrin receptor.

**TABLE 3:** Factors associated with BRINDA adjusted sTfR and ferritin deficiencies among adolescent boys aged 10–19 years in Nepal.

Characteristic	Unadjusted OR (95% CI)	*p*	Adjusted OR (95% CI)	*p*
sTfR				
Ethnicity (Caste)				
Brahmin/Chettri	1.00		1.00	
Dalit	1.37 (0.63, 3.02)	0.422	1.36 (0.62, 2.89)	0.456
Janajati	2.34 (1.39, 3.94)	0.002	2.22 (1.29, 3.82)	0.004
Others	1.33 (0.54, 3.28)	0.528	1.28 (0.52, 3.15)	0.594
Age (continuous)	0.89 (0.82, 0.98)	0.012	0.91 (0.83, 0.99)	0.034
Consumed eggs				
Yes	1.00		1.00	
No	4.75 (1.41, 15.91)	0.012	4.35 (1.24, 15.24)	0.022
Ferritin				
Geographical region				
Western	1.00		1.00	
Far‐Western	7.02 (1.52, 32.36)	0.013	6.82 (1.50, 31.06)	0.014
Central	7.44 (1.80, 30.79)	0.006	7.66 (1.84, 31.92)	0.006
Mid‐Western	2.83 (0.67, 11.91)	0.154	2.93 (0.67, 12.88)	0.152
Eastern	2.49 (0.59, 10.46)	0.209	2.35 (0.57, 9.68)	0.232
Age (continuous)	0.82 (0.72, 0.94)	0.006	0.83 (0.72, 0.95)	0.007

Abbreviations: OR, odds ratio; sTfR, serum transferrin receptor.

The mean ferritin concentration among adolescent girls aged 10–19 years was 35.2 μg/L (SD ±22.3), with 18.0% classified as iron‐deficient. sTfR concentration was 6.8 mg/L (SD ±4.5), and 13.8% of girls reported sTfR deficiency. The mean vitamin A concentration was 1.2 μmol/L (SD ±0.3), with a deficiency prevalence of 4.4% and the mean serum concentration of folate was 499.8 ± 241.8 nmol/L, and the prevalence was 16.2%.

### 3.3 Factors Associated With Ferritin. sTfR, Vitamin A, and RBC Folate Deficiencies

#### 3.3.1 Boys

Table 3 presents the factors associated with iron deficiency (sTfR, ferritin concentrations) among adolescent boys. Adolescent boys from the Janajati ethnic group were significantly more predisposed to being iron insufficient (sTfR levels) than their counterparts from the Brahmin/Chettri ethnic group [AOR: 2.22; 95% CI: (1.28, 3.82)]; and the likelihood of iron deficiency (sTfR concentrations) was significantly higher among adolescent boys who did not consume eggs compared to those who did [AOR: 4.35; 95% CI: (1.24, 15.24)]. Additionally, the likelihood of being iron deficient (sTfR levels) increased with decreased age of the adolescent boy [AOR: 0.91; 95% CI: (0.83, 0.99)]. The likelihood of being iron deficient (ferritin concentrations) was significantly higher among adolescent boys who resided in the central region compared to their counterparts who resided in the western region [AOR: 7.66; 95% CI: (1.84, 31.92)]; and the likelihood of iron deficiency (ferritin concentrations) increased with decreased age the adolescent boy [AOR: 0.83; 95% CI: (0.72, 0.95)].

#### 3.3.2 Girls

Adolescent girls from the eastern region were significantly more predisposed to folate deficiency compared with those from the western region [AOR: 4.02; 95% CI: (2.41, 6.69)] (Table 4). The likelihood of being folate deficient was significantly higher among adolescent girls from the other ethnic groups other than the Janajati and Dalit tribes, compared with those from the Brahmin/Chettri ethnic group [AOR: 2.71; 95% CI: (1.50, 4.90)]. Adolescent girls with low or normal BMI had higher odds of ferritin deficiency compared with those who had a BMI ≥ 25 kg/m^2^. The likelihood of vitamin A deficiency was significantly higher among adolescent girls from the Dalit caste group compared with those from the Brahmin/Chettri group [AOR: 3.91; 95% CI: (1.39, 10.96)]. The likelihood of vitamin A deficiency increased with decreased age of the adolescent girl [AOR: 0.74; 95% CI: (0.65, 0.85)]. Furthermore, adolescent girls who did not consume nuts or seeds or any food made from nuts or seeds were significantly more predisposed to being vitamin A deficient compared with those who did [AOR: 8.50; 95% CI: (1.02, 70.66)].

**TABLE 4:** Factors associated with ferritin, sTfR, RBC folate, and vitamin A deficiencies among adolescent girls aged 10–19 years in Nepal.

Characteristic	Unadjusted OR (95% CI)	*p*	Adjusted OR (95% CI)	*p*
Ferritin				
Geographical region				
Western	1.00		1.00	
Far Western	1.72 (1.08, 2.75)	0.023	1.82 (1.10, 3.01)	0.020
Central	1.53 (1.03, 2.27)	0.035	1.46 (0.96, 2.22)	0.077
Mid‐Western	1.01 (0.66, 1.54)	0.958	1.04 (0.66, 1.64)	0.855
Eastern	1.57 (1.02, 2.42)	0.041	1.70 (1.07, 2.64)	0.024
Weight (continuous)	1.04 (1.02, 1.05)	< 0.001	1.05 (1.03, 1.08)	< 0.001
BMI				
25+	1.00		1.00	
19–25	5.83 (1.32, 25.72)	0.021	11.52 (2.95, 45.07)	0.001
≤ 18.5	2.79 (0.65, 11.94)	0.163	10.81 (2.80, 41.71)	0.001
sTfR				
Ethnicity (Caste)				
Brahmin/Chettri	1.00		1.00	
Dalit	1.54 (0.97, 2.43)	0.064	1.50 (0.80, 2.81)	0.201
Janajati	1.67 (1.06, 2.62)	0.028	1.82 (1.04, 3.20)	0.038
Others	1.29 (0.65, 2.57)	0.459	0.90 (0.49, 1.66)	0.738
Ecological zone				
Mountain	1.00		1.00	
Hill	1.03 (0.56, 1.91)	0.921	1.23 (0.70, 2.15)	0.468
Terai	1.94 (1.05, 3.59)	0.035	3.01 (1.72, 5.28)	< 0.001
BIS (mg/kg) (continuous)	0.59 (0.54, 0.64)	< 0.001	0.58 (0.53, 0.62)	< 0.001
Consumed eggs				
Yes	1.00		1.00	
No	2.15 (1.20, 3.86)	0.011	3.15 (1.48, 6.69)	0.003
Folic acid/RBC folate				
Geographical region				
Western	1.00		1.00	
Far‐Western	1.61 (0.87, 2.99)	0.129	1.38 (0.76, 2.50)	0.285
Central	2.58 (1.29, 5.18)	0.008	2.65 (1.41, 4.97)	0.003
Mid‐Western	1.87 (1.05, 3.32)	0.034	1.94 (1.10, 3.44)	0.024
Eastern	3.28 (1.86, 5.79)	< 0.001	4.02 (2.41, 6.69)	< 0.001
Ethnicity (Caste)				
Brahmin/Chettri	1.00		1.00	
Dalit	1.61 (0.97, 2.66)	0.066	1.84 (1.13, 2.99)	0.015
Janajati	1.08 (0.69, 1.68)	0.728	1.38 (0.91, 2.09)	0.133
Others	1.94 (1.14, 3.33)	0.016	2.71 (1.50, 4.90)	0.001
Vitamin A				
Ethnicity (Caste)				
Brahmin/Chettri	1.00		1.00	
Dalit	4.47 (1.57, 12.7)	0.006	3.91 (1.39, 10.96)	0.010
Janajati	1.61 (0.58, 4.45)	0.354	1.39 (0.50, 3.84)	0.520
Others	3.77 (1.34, 10.64)	0.013	3.39 (1.23, 9.37)	0.019
Age (continuous)	0.74 (0.65, 0.84)	< 0.001	0.74 (0.65, 0.85)	< 0.001
Consumed nuts and seeds or any food from nuts or seeds				
Yes	1.00		1.00	
No	10.28 (1.27, 82.86)	0.029	8.50 (1.02, 70.66)	0.048

Abbreviations: OR, odds ratio; sTfR, serum transferrin receptor.

## 4 Discussion

The current study utilized data from the 2016 NNMSS to conduct a multivariable analysis that adjust for clustering, stratification, and sampling weights to assess the sociodemographic factors and diet habits associated with iron deficiency and vitamin A deficiency among Nepalese adolescent boys and girls aged 10–19 years. Majority of the participants resided in rural areas. After controlling for potential confounding factors, it was found that younger adolescents were particularly vulnerable, with increased odds of iron and vitamin A deficiencies observed across both sexes. Adolescents who did not consume sufficient meat, eggs, fruits, or foods made from nuts and seeds had significantly higher odds of deficiency, low to normal BMI was also associated with increased odds of ferritin. This study also found that regional disparities were evident. Adolescents from the eastern region, the Terai ecological zone, and the central region experienced higher odds of sTfR levels compared with those from western areas. Ethnic inequalities were also observed and among boys, those from the Janajati ethnic group were more likely to be iron insufficient (sTfR) compared with Brahmin/Chettri counterparts. Among Adolescent girls, those from the Dalit caste group were at greater odds of vitamin A deficiency, while girls from Eastern and far western had higher odds of folate deficiency relative to Western girls. Overall, this study demonstrated that adolescent micronutrient deficiencies such as iron, vitamin A, and folate in Nepal are shaped by factors including age, diet, ethnicity, body composition, and geographic location.

In this current study, we found that sTfR and ferritin increase as adolescent boys' age decreases, indicating that late adolescent boys are less prone to micronutrient deficiency including sTfR and ferritin. This finding is consistent with a finding from a past study in India (Deka et al. 2015), which could be explained by the fact that early adolescents are more vulnerable to malnutrition than their late counterparts (Deka et al. 2015). More detailed research is needed to establish the relationship between early adolescent boys, sTfR, and ferritin deficiencies.

In Nepal, as in other countries of the world, there have been very few studies on micronutrient deficiencies among adolescents compared to other groups, such as women and children (Baral and Onta 2009). To the best of our knowledge, this is the first study on micronutrient deficiency among adolescent girls and boys in Nepal. It is important to undertake a study on sTfR and ferritin deficiencies in girls and boys because such studies would have a crucial impact on health issues such as cognitive and physical growth and development.

Adolescent girls who lived in the Terai ecological zone of Nepal were significantly more predisposed to iron deficiency (sTfR level) than those who lived in the Mountain ecological zone. The association of iron deficiency including ferritin with geographic location has also been revealed in previous studies (Kattel 2016; Preziosi et al. 1997; Organization WH, United Nations Children's Fund/United Nations University 2001). Similar studies on iron deficiency in Nepal (Ford et al. 2022; Chalise et al. 2018) reported that despite the fertile lands of the Terai region, poverty and income disparities are higher than in other regions of Nepal (Pokharel 2015), resulting in food insecurity and limited consumption of a variety of foods. Further, poor sanitation in the Terai region and the practice of walking barefoot lead to the transmission of helminths, thus inhibiting the absorption of nutrients (Ford et al. 2022; Chalise et al. 2018).

We found that adolescent girls and boys who did not consume eggs were significantly more predisposed to iron deficiency (sTfR level) than those who did. Our finding is consistent with findings from a study in Vietnam, which revealed that inadequate consumption of eggs is positively correlated with iron deficiency in pregnant women (Aikawa et al. 2006). The iron content in eggs is low; however, the bioavailability of iron in eggs is enhanced when consumed with foods high in ascorbic acid (Thompson et al. 2010). Adolescent boys from the Janajati ethnic group were found to be significantly more predisposed to iron deficiency (sTfR level) compared with those from the Brahmin/Chettri ethnic group. This highlights an association between iron deficiency and dietary patterns among different ethnicities in Nepal. Our finding is consistent with findings from previous studies (Das et al. 2000; Sengupta et al. 2002; Balgir et al. 2003). The finding may be attributed to the fact that populations of such iron deficiency‐prone ethnic groups are mostly exposed to low bioavailability of iron, as their staple food is mostly cereal‐based with little dietary diversity (Chatterjee et al. 2011).

Adolescent girls are at higher odds of iron deficiency due to the low availability and access to a nutrient‐rich diet in addition to the onset of menarche (Gautam and Sharma 2011). We found that residence in the Terai ecological zone was positively associated with iron deficiency (sTfR as a biomarker) in adolescent girls. Additionally, we found that the odds of iron deficiency (ferritin as a biomarker) were positively associated with adolescent girls from the Far‐western region of Nepal. Similar findings have been made in previous studies that revealed that geographic location is associated with iron deficiency and is normally higher in rural areas than in urban ones (Preziosi et al. 1997; Organization WH 2001). Low education, early marriage, and childbearing during adolescence may also adversely affect the nutritional status of adolescent girls (Chalise et al. 2018). Gender‐based food discrimination practices and food distribution discrimination during menstruation cycle practices among different castes in Nepal also have tremendous impacts on food availability among girls and women (Bishokarma and Amir 2014). A study in Uganda found the prevalence of iron deficiency to be higher in one sector (the Bulange sub‐county) than in other areas (the Namutumba and Magada sub‐counties). These differences may be an indication of the fact that some regions are better off in terms of nutritional education, availability of appropriate and adequate foods, or socioeconomic resources. Further studies should be carried out to examine the differences to understand why adolescent girls in some regions are more prone to the biomarkers of micronutrient deficiencies such as sTfR, ferritin, and folic acid/RBC folate than those from other regions. Appropriate interventions could target those disadvantaged regions to improve the burden of micronutrient deficiencies in them.

Our study revealed that adolescent girls with low and normal BMI had a significantly higher odds of being iron deficient (ferritin) than those with BMI of 25 kg/m^2^ or higher. Our general observation was that there was a significantly higher odds of iron deficiency for low BMI among adolescent girls. Our finding is similar to a study done in India (Bentley and Griffiths 2003) which also found a higher prevalence of iron deficiency (ferritin level) among women with low and normal BMI compared to women who were overweight. We suspect that poor dietary patterns with inadequate iron intake or other micronutrient deficiency that interferes with iron metabolism may be involved, however, we are unable to determine or assess this with the current data and hence, further research is needed to examine how low and normal BMI are associated with ferritin levels.

Adolescent girls from the Janajati ethnic group had a significantly higher odds of iron deficiency (folic acid/RBC folate and sTfR) compared with those from the Brahmin ethnic group. This finding is consistent with a similar study that also found a high prevalence of iron‐deficiency anemia, as indicated by biomarkers for iron, including folic acid/RBC folate, serum folate and sTfR levels among Janajati women (Agho, Chitekwe, Sahiledengle, et al. 2024; Sharma et al. 2020). The susceptibility of iron deficiency among girls and women of Janajati ethnic group could be due to but not limited to illiteracy, social discrimination, caste‐based discrimination, inaccessibility to health facilities, malnutrition, deprivation of economic opportunities and other traditional restrictive beliefs and practices among this ethnic minority group (Gautam and Sharma 2011; Sharma et al. 2020). A similar finding was made in a study in Benin, where women from the Boo ethnic group were a risk factor to iron deficiency including folic acid/RBC folate and sTfR as biomarkers for iron (Alaofè et al. 2017). This finding highlights differences by ethnicity in the odds of iron deficiency and serves as a significant indicator of unequal distribution in nutrition in Nepal. Therefore, further research is needed on micronutrient deficiencies among and within various ethnic settings of Nepal.

We found that the nonconsumption of eggs by adolescent girls is significantly associated with sTfR deficiency. This is consistent with a previous study in Vietnam (Aikawa et al. 2006), which buttresses the fact that adequate protein intake plays a key role in preventing iron deficiency, including sTfR as a biomarker for iron. In Nepal, young people are encouraged to rear chickens, among other small animals, as part of upholding the food security of households; although many households practice poultry, they prefer to sell the chickens and eggs rather than directly consume the eggs.

In this study, it was observed that adolescent girls who did not consume nuts, seeds, or foods were significantly more likely to be vitamin A deficient compared with those who did consume these foods. This finding is consistent with a study by O'Neil et al. (2015), which reported that consumers of tree nuts significantly had a lower percentage of the population below the Healthy Eating Index‐2005 (HEI‐2005) component scores for vitamin A. However, this association should be interpreted with caution, as cross‐sectional analyses of dietary patterns may be imprecise due to potential sparse‐data bias, particularly when sample sizes are small in vitamin A. Such limitations may lead to overestimation or underestimation of the odds ratios and further restrict causal inference in cross‐sectional analyses.

A major strength of the current study was that it used a dataset from the Nepal National Micronutrient Survey, which is a population‐based and nationally representative population, including a high response rate of over 98% for both sexes. The first nationally representative, population‐based NNMSS examining deficiencies in essential vitamins and micronutrients among adolescents in Nepal. Therefore, results from the study may be generalisable to a larger. However, there were limitations to this study. Firstly, because the dataset used was cross‐sectional in design, only associations could be reported, and causality between dependent and independent variables could not be ascertained. Secondly, measuring extra laboratory variables such as sTfR could have resulted in increased detection of iron deficiency. Thirdly, the clinical consequences of iron and vitamin A deficiencies among adolescent boys could not be determined, as health outcomes were not included in the dataset used. Fourthly, using ferritin concentrations for evaluating iron status may be misleading, as these concentrations are prone to diurnal rhythms, which increase after the iron‐containing foods are ingested (Sauberlich 1999). Finally, this study relied on self‐reported dietary intake data, which may not accurately reflect participants' habitual diet. Such measures are subject to recall bias and may be influenced by day‐to‐day variation in food consumption, potentially leading to misclassification of usual nutrient intake (Subar et al. 2015; Shim et al. 2014; Tooze et al. 2010).

## 5 Conclusion

Iron deficiency including folic acid/RBC folate and sTfR are global public health problems and were found to be prevalent among adolescent boys and girls in Nepal. The findings of this study revealed the non‐biological factors associated with these diseases. These factors included residence in poor households and central regions, the Janajati ethnic group, and nonconsumption of meat and eggs. The findings of this study revealed that the common factors associated with anemia and iron deficiency among adolescent boys and girls in Nepal were residing in poor households and in the central regions, being from the Janajati ethnic group, and nonconsumption of meat and eggs. The Nepalese government and other stakeholders should also ensure equitable distribution of nutritional programs among the various deprived regions and zones, and for that matter, the ethnic groups. There should be a robust continuation of the current Nutrition policy and strategy that targets micronutrients especially among adolescent boys and girls.

## Author Contributions

Conceptualization: K.E.A., S.C., and L.N.P. Methodology: K.E.A. and S.C. Validation: K.E.A., S.C., L.N.P., and B.S. Formal analysis: K.E.A. and S.C. Investigation: N.P., S.K.S., and K.E.A. Resources: N.P., S.K.S. Writing – original draft preparation: K.E.A., S.C., and L.N.P. Writing – review and editing: K.E.A., S.C., L.N.P., B.S., S.R., N.P., S.K.S., A.R. Supervision: K.E.A. and S.C. Data acquisition: N.P., S.K.S., A.R. All authors have read and agreed to the published version of the manuscript. K.E.A. agreed to be accountable for all aspects of the work.

## Funding

The authors have nothing to report.

## Conflicts of Interest

The authors declare no conflicts of interest.

## Data Availability

The data that support the findings of this study are available on request from the corresponding author. The data are not publicly available due to privacy or ethical restrictions.
